# ‘Whole Organism’, Systems Biology, and Top-Down Criteria for Evaluating Scenarios for the Origin of Life

**DOI:** 10.3390/life11070690

**Published:** 2021-07-14

**Authors:** Clifford F. Brunk, Charles R. Marshall

**Affiliations:** 1 Department of Ecology and Evolutionary Biology, University of California Los Angeles, Los Angeles, CA 90095-1606, USA; 2 Department of Integrative Biology, Museum of Paleontology, University of California, Berkeley, CA 94720-4780, USA

**Keywords:** origin of life scenarios, alkaline hydrothermal vent microchambers, prebiotic chemistry, RNA world, Initial Darwinian Ancestor (IDA), LUCA, chemiosmosis, ATP synthase, Archaea, Bacteria

## Abstract

While most advances in the study of the origin of life on Earth (OoLoE) are piecemeal, tested against the laws of chemistry and physics, ultimately the goal is to develop an overall scenario for life’s origin(s). However, the dimensionality of non-equilibrium chemical systems, from the range of possible boundary conditions and chemical interactions, renders the application of chemical and physical laws difficult. Here we outline a set of simple criteria for evaluating OoLoE scenarios. These include the need for containment, steady energy and material flows, and structured spatial heterogeneity from the outset. The Principle of Continuity, the fact that all life today was derived from first life, suggests favoring scenarios with fewer non-analog (not seen in life today) to analog (seen in life today) transitions in the inferred first biochemical pathways. Top-down data also indicate that a complex metabolism predated ribozymes and enzymes, and that full cellular autonomy and motility occurred post-LUCA. Using these criteria, we find the alkaline hydrothermal vent microchamber complex scenario with a late evolving exploitation of the natural occurring pH (or Na^+^ gradient) by ATP synthase the most compelling. However, there are as yet so many unknowns, we also advocate for the continued development of as many plausible scenarios as possible.

## 1. Introduction

Explaining the origin of life on Earth (OoLoE) has proven difficult, primarily because even the simplest organisms today have an enormously complex hierarchical organization, with intricate interdependencies between their various internal functions, the pathways that enable them, and their spatially and temporally changing external environments. These difficulties are compounded by the fact that life probably arose by 4 billion years ago on a very different Earth from now, and that we only have data from one origin. Nonetheless, the appeal of the problem, fueled by its inherent difficulty, has led to both decades-old divisions [1,2] and a huge literature—a Google Scholar search from 2001–2020 for ‘origin of life’ yields 25,400 results. While we are not concerned here with life elsewhere in the universe, we note that the burgeoning field of astrobiology has also injected enormous energy into the study of the origin of life, with 119,000 Google Scholar search results from 2001–2020. 

### 1.1. The Value of Scenarios

While piecemeal experimental data are crucial for advancement [1], for example Miller’s [3,4] demonstration of the ease (in principle) of prebiotic synthesis and the discovery of ribozymes [5,6], ultimately we want to know how piecemeal advances fit into more cohesive narratives for the origin of life. Such scenarios are, of course, speculative, but they are valuable in focusing future research, providing contexts for framing the diverse literature, and in the end one of our goals is to develop a convincing scenario that does indeed explain the origin of life and the path from there to life on Earth as we currently know it.

### 1.2. The Range and Scope of Proposed Initial Venues and Accompanying Scenarios

A wide range of scenarios has been proposed, with varying degrees of scope. Most begin with a specific venue for the initiation of prebiotic chemistry, although some advocate for the role of multiple environments in life’s origin [7,8,9,10]. Proposed beginning venues include marine alkaline hydrothermal vents, either within microchambers [11,12,13,14,15,16] or iron (oxy)hydroxide (green rust) mounds [2,17], hydrothermally/volcanically charged sediments [18,19], terrestrial geothermal hot springs [20] including at their edges [21,22], within the pores of pumice rafts [23,24], between layers of micas [25], on clay surfaces [26] or greenalite nanoparticles [27], and even within the atmosphere as the key venue for initial biosynthesis fueled by a major meteorite impact [10]. Many of these have been recently summarized and evaluated [28], and we do not undertake a comprehensive analysis of the proposed starting venues here. 

We note that a standard default venue for the origin of life is Darwin’s warm little pond, described in his letter to J.D. Hooker in 1871 [29]. However, Darwin’s accompanying text indicates that his purpose was not to propose a venue for the origin of life (nor does it appear that he felt it was possible to do so at the time [30]), but to point out that wherever life originated that it was unlikely to originate in that setting again because the now present life would consume the needed resources: “*It is often said that all the conditions for the first production of a living being are now present.—But if (and oh what a big if) we could conceive in some warm little pond with all sorts of ammonia and phosphoric salts,—light, heat, electricity … present, that a protein compound was chemically formed, ready to undergo still more complex changes, at the present such matter would be instantly devoured, or absorbed, which would not have been the case before living creatures were formed”*. C. R. Darwin 1871.

Given a venue, some work back in time to explore how the needed raw materials were supplied (e.g., [22]), while most focus on the transition from the first stages of prebiotic chemistry to one or more of the stages from the emergence of early metabolism, self-replication, the Last Universal Common Ancestor (LUCA), and the living biota. The most comprehensive scenarios have been developed for the alkaline hydrothermal vent microchamber [16] and green rust mound [17] venues, hydrothermally charged marine sediments [18,31], and associated with terrestrial geothermal hot springs [20,21]. We note that the more comprehensive the scenario, the more room there is for different variants, and so each scenario typically consists of a set of closely related variant scenarios. Thus, for example, for the alkaline hydrothermal vent microchamber scenario, some emphasize the importance of the pH difference between the vent flux and the open ocean [32], while others focus more on the chemical energy (H_2_) in the vent flux [12]. 

### 1.3. Criteria for Evaluating Scenarios

Most of the debate over OoLoE scenarios (quite sensibly) centers on the plausibility of the (bio)chemistry needed or implied. However, a pervasive difficulty of this bottom-up approach is the lack of constraints on the chemical and physical conditions, for example, the temperature, pH, source materials, the nature of the containment, the identity and importance of co-occurring chemical species, and to what extent spatial and temporal heterogeneity in these variables played a role. We do not pretend to solve this complex problem here, nor do we deal with many of the difficult issues, for example, the emergence of homochirality [33], how in detail the first metabolism arose, or modern membrane systems [34], to name just a few. Instead, we take a step back and discuss some over-arching criteria that, especially when considered jointly, we hope will aid our attempt to dissect out the strengths and weaknesses of various scenarios, that is, that might help us see the forest for the trees. 

### 1.4. Some Terminology

**Figure 1:**
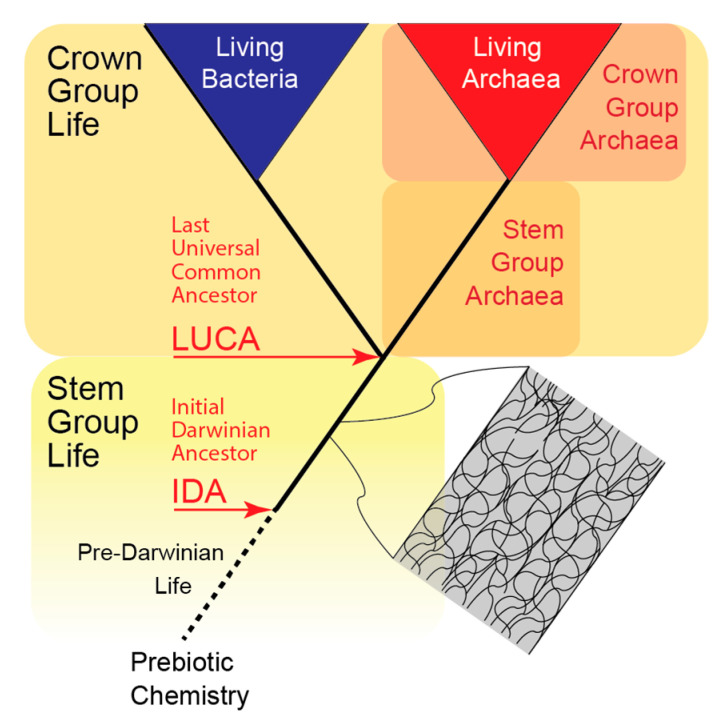
Terminology used in this paper. ‘Crown Group Life’ refers to the most recent common ancestor of all living things (LUCA) and all its descendants (regardless of whether they are extinct or not). ‘Stem Group Life’ refers to all living things (now extinct) that predate LUCA, including the Initial Darwinian Ancestor (IDA) [37] back to the hard-to-define transition between prebiotic chemistry and Pre-Darwinian Life, which consists of systems that might be viewed as being alive, but not yet capable of reproduction. The terms crown and stem can be applied to any lineage, as shown here for the Archaea. The grey inset is meant to convey the likelihood that there was extensive exchange of information and molecules between entities that might be viewed as being alive throughout all the early phases of life’s history. Note that we have not included the eukaryotes in this figure.

To aid our discussion we use the terms crown and stem groups (Figure 1), drawn from the paleontological literature [35,36] to capture the relationship of the living biota to now extinct life forms. The crown group corresponds to the LUCA and all its descendants, whether extant or extinct. Stem-group life refers to everything before LUCA, including the Initial Darwinian Ancestor (IDA) [37] and all the way back to the earliest point at which the notion of ‘aliveness’ might be applied (see Section 2). Entities that might be viewed as alive but cannot yet reproduce we call pre-Darwinian Life. Anything before that we refer to as prebiotic chemistry. By definition, all members of the stem group are extinct. 

In this paper when we say the origin of life, we mean it in several senses, from the hard-to-define transition from prebiotic chemistry to pre-Darwinian life, to the origin of the IDA, the origin of the LUCA (defined by the divergence of stem group Bacteria from stem group Archaea), to the origin of crown group Bacteria and Archaea.

## 2. ‘Whole Organism’ Criteria

In today’s world the entities that we typically identify as being organisms share the common properties of: (1) being spatially well-circumscribed; (2) being dependent on (relatively) steady flows of energy and materials; (3) having complex interiors; (4) having most of their constituent parts repeatedly replaced molecule by molecule, a consequence of (1)–(3) [38]. We note that entities with properties (1)–(4) might typically be judged as being alive. Thus, while organisms also reproduce, and while reproduction is key to evolutionary change [39,40], reproduction, per se, is not required to judge something as being alive. Thus, while many definitions of life include reproduction and the capacity for evolution as criteria [41], and thus a Darwinian component, for example, NASA’s working definition of life as a “self-sustaining chemical system capable of Darwinian evolution” [42,43], we posit that being alive is a more encompassing term, and argue below that being alive in this sense predated life capable of Darwinian evolution. 

Note we are not trying to be comprehensive in our list of attributes of what it means to be alive, nor are we trying to define life or ‘aliveness’ here. Instead we are simply making explicit our sense that these properties were most likely present prior to the very beginning of life. That is, that containment that admitted a steady flow of materials and energy into and out of the contained space, situated in an environment where those steady flows were reliably available and long-lived, were present from the outset (Table 1, criterion 1). 

We note that most of the venues listed above are predicated on this supposition, which means this criterion used in isolation has limited discriminatory power. Nonetheless, the criterion also means that scenarios that only concentrate on prebiotic synthesis of one or a few key molecules without reference to these ‘wholeness’ criteria are very incomplete. 

### Probability of Life

Another way of assessing origin of life scenarios is simply to ask how likely the origin of life was under each scenario, under the premise that all else being equal, the higher the probability the more likely the scenario (Table 1, criterion 2).

## 3. Systems Biology Criteria

The notion that ‘wholeness’ was present from the outset leads to two further criteria that stem from a systems biology perspective. 

### 3.1. Integration Began from the Outset

First, all organisms today exhibit sophisticated integration among their component parts, whether it is among the various organs in animals, to the various interconnected pathways that constitute cellular metabolism in all cells. From an evolutionary standpoint, the fact that integrated complexity had to arise piecemeal [44], leads to our next criterion, that integration began at the inception of pre-Darwinian life, within the initial spaces that contained it (Table 1, criterion 3). 

### 3.2. Spatial Heterogeneity—Multi-Pot Rather Than Single-Pot Prebiotic Chemistry

The second systems biology criterion follows from the observation that integrated function is only possible via structured spatial heterogeneity, that is, that spatial heterogeneity is crucial to cellular function. The importance of this heterogeneity has been made especially clear with the discovery of molecular condensates, protein delimited compartments that function at many spatial scales within eukaryotic cells [45], with increasing focus now on prokaryotes [46]. The importance of spatial heterogeneity has also been noted in the discussion of the prebiotic generation of macromolecule precursors [47,48,49,50]. These observations lead to the supposition that spatial chemical inhomogeneity was continually present from the earliest phases of life’s development (Table 1, criterion 4). 

## 4. Top-Down Criteria and the Principle of Continuity

The criteria outlined above alone do not offer a great deal of discriminatory power, unless augmented with top-down data.

### 4.1. The Principle of Continuity Connects Bottom-Up with Top-Down Data

Most OoLoE scenarios (quite sensibly) begin with the abiotic world and build towards first life, the bottom-up approach. In contrast, top-down approaches begin with organisms alive today and try to work back to their simpler extinct predecessors, specifically LUCA, typically using phylogenetic analysis [51,52]. However, gene-by-gene, or gene family-by-gene family, or functional system-by-functional system phylogenetic approaches to top-down analysis have proven challenging [1,53,54]. The difficulties include the erosion of the signal from sequential vertical transmission of evolutionary novelties by horizontal transfer, phylogenetic noise (the difficulty in inferring correct topologies), loss of function, and subsequent evolutionary change that has over-printed initial functions and/or structures and/or sequences. Moreover, even if executed robustly, top-down data has limited utility for inferring the temporal sequence of events prior to (LUCA) [42].

Nonetheless, at some point we must connect the features of first life with the properties of organisms living today, sometimes termed the Principle of Continuity [21,55,56,57]. Thus, one can ask of bottom-up scenarios to what extent they are compatible with, or better explain, information gleaned from top-down data. Another way of casting this is to ask how information gleaned from the phylogenetic distribution of traits among the living taxa helps inform our search for convincing scenarios for the origin of life. Despite the difficulties in conducting top-down analysis, some clear signals do emerge. For example, the fact that Bacteria and Archaea have different DNA replication systems [58] but use the same translation system implies that translation evolved before the comprehensive transfer of information storage to DNA. We now outline three implications (of a no doubt longer list) for origin of life scenarios that emerge from this line of reasoning. 

### 4.2. Early Versus Late Cellularization

One of the enigmas of modern biology is the puzzling differences between Archaea and Bacteria. They share the intricate details of translation, indicating that LUCA had already established protein synthesis with the genetic code, the ribosome, the full complement of tRNAs, etc. Yet, Bacteria and Archaea employ different molecules in their cell membranes and cell walls, where bacterial membranes consist of phospholipids with fatty acid chains linked via ester bonds to glycerol-3-phosphate, while archaeal phospholipids have isoprenoid chains linked via ester bonds to glycerol-1-phosphate [59,60,61]. Their cell walls also have different compositions, the bacterial cell wall consisting of peptidoglycan (murein) [62], whereas Archaea employ an S-layer composed of a monomolecular layer of identical proteins or glycoproteins [63]. 

Taken at face value, these data suggest that cellularization, in the sense of being encapsulated in such a way that enabled full autonomy, evolved after LUCA [50,64] (Table 1, criterion 5). Thus, top-down data combined with the Principle of Continuity implies late cellularization.

Now, to be clear, while the top-down data indicate a post-LUCA origin of full cellular autonomy, the ease with which vesicles form spontaneously [65], strongly suggests that the molecular constituents of vesicles, and thus the molecular constituents of protocells, were almost certainly part of the pre-Darwinian complexity. That is, it seems likely that membrane-like structures were an integral part of the pre-Darwinian world, well before there was fully autonomous mobility.

### 4.3. Additional Evidence for Late Cellularization—Different Locomotory Structures

In support of the supposition that the Bacteria and Archaea independently cellularized, we note that they have non-homologous locomotory structures, flagella in Bacteria and archaella in Archaea [66], where for example, Bacteria use proton motive force to drive the rotation of their flagella, while Archaea use ATP to drive the rotation of their archaella. In both lineages it appears that adhesins were independently co-opted for their locomotory structures [66], suggesting each lineage first attached to the substrate before gaining the capacity for locomotion [66].

### 4.4. Analog Versus Non-Analog Properties and the Principle of Continuity

We here define analog properties, for example, pathways of biosynthesis or molecular compositions, as those that had their origins prior to LUCA that persist to the present. Non-analog properties are initial properties that have been replaced in crown group life. Thus, for example, the hypothesis that polynucleotides were initially synthesized from HCN using UV light and wet–dry cycles [22] is a non-analog process, using non-analog energy and material sources. Similarly, the hypothesis that life originated within microchambers in alkaline hydrothemal vents involves non-analog containment, which requires a transition to analog cell membranes and cell walls [50,64].

Given the Principle of Continuity from the initial prebiotic conditions to life today, all else being equal, the principle of parsimony (Occam’s razor) supports the favoring of scenarios with the fewest number of non-analog to analog transitions [67] (Table 1, criterion 6). However, having said this, we agree that there is no a priori reason to suppose that any of life’s functions began as direct analogs of the processes of crown group life (for example, see [68] for a review in the context of nucleotide synthesis)—we are simply saying that, all else being equal, a scenario that begins with an analog process should be favored over one that begins with a non-analog process [69]. 

### 4.5. Pre-Darwinian Complexity—Implications from Top-Down Systems Biology 

We here draw upon two recent top-down analyses that point to the likelihood of significant integrated complexity in the network of pre-Darwinian pathways prior to prevalent ribozymes, that is, prior to the Initial Darwinian Ancestor (IDA). The first study notes that many of the living metabolic pathways could be run without enzymes and ribozymes, using instead minerals, metal ions, and small molecules as catalysts [69]. These pathways include the building and breakdown of ketoacids, sugars, amino acids and ribonucleotides, and functional equivalents of the acetyl-CoA pathway, (r)TCA cycle, gluconeogenesis/glycolysis, and pentose phosphate pathway [69].

The second is the targeted search [70,71,72] for autocatalytic cores [73] embedded within current prokaryotic biochemical networks, specifically reflexively autocatalytic food-generated networks (RAFs)—self-sustaining networks that collectively catalyze all their own reactions, without the need for enzymes or ribozymes [74]. With these top-down data they identify a connected network of 172 reactions involving 175 metabolites, enriched in metal catalysts and carbon-metal bonds. 

Taken together, these studies suggest that pre-Darwinian life was remarkably complex, with all the major classes of molecule playing their roles in life’s early metabolism (Table 1, criterion 7).

### 4.6. Implications for the “RNA-World”—The “Plus-RNA-World”

The presence of considerable pre-ribozymal/pre-enzymatic complexity strongly supports the supposition that the beginning of Darwinian Life, the emergence of the IDA, while dependent on the origination of self-replicating ribozymes, emerged from an already complex and well integrated system [69,74,75,76]. To reflect this likelihood, we here propose that the “RNA-World” be called the “plus-RNA-World” to make explicit that the role of RNA was added onto an already complex functioning metabolism. 

## 5. The Role of Chemical and Physical Principles 

Table 1 summarizes our proposed set of interconnected ‘whole organism’, systems biology, and top-down criteria for evaluating OoLoE scenarios. Beyond these criteria, for any and all scenarios, the chemistry and physics obviously also has to work [69]. While the application of the laws of chemistry and physics is indispensable, and has proven invaluable in some instances, for example, in the general agreement that the black smokers are too hot and too acidic to have served as the cradle of life, their application is often tricky—the dimensionality of non-equilibrium chemical systems, from the range and combination of possible boundary conditions (temperature, pressure, chemical constituents, and temporal and spatial heterogeneity) to the range of chemical interactions that may or may not have occurred renders the sole application of chemical and physical principles difficult. 

For example, the fact that today mid-ocean ridges represent phosphorous sinks [77] has led some to seriously doubt that alkaline hydrothermal vents could have been the venue for life’s origin [78]. However, a systems biology approach suggests an early phosphate-free nonenzymatic biochemistry [79], which thus supports the origin of life in a phosphorous depleted environment, while the discovery of abundant nanometer apatite (Ca_10_(PO_4_)_6_(OH,F,Cl)_2_) crystals in 2.46 to 3.46 Ga year old banded iron formations (BIFs) and cherts suggests there may have been, in fact, dissolved phosphate available from hydrothermal fluxes prior to the rise of oxygen [80]. 

Some have also argued that protocell self-assembly is unfavorable in high salinity, suggesting marine environments could not have served as the cradle of life (for example, see [81,82]). However, others have provided evidence that protocell self-assembly can occur in an alkaline hydrothermal vent setting (for example, see [83]).

Similarly, consideration of the composition of the cell cytoplasm suggests that life originated in a habitat with a high K^+^/Na^+^ ratio, raising doubts about a marine origin of life, given the inferred high concentration of Na^+^ in the anoxic primordial oceans [20]. However, the recent discovery of alkaline hydrothermal vents in the coastal waters of Iceland with an internal concentration of Na^+^ 1–2 orders of magnitudes lower than seawater [84] indicates that high K^+^/Na^+^ ratios can be found in marine settings. Moreover, the presence of steep H^+^ and Na^+^ gradients in these vents also suggests the possibility, under the alkaline hydrothermal vent microchamber scenario, that ATP synthase made use of a Na^+^ gradient from its inception, which would solve some of the conundra that emerge if only an H^+^ gradient was available [16,32,59,85,86,87,88]. The world is a very complex place—certainty is often hard to find even with the power of chemical and physical laws.

## 6. Value-Added Criterion

Finally, we note that a hallmark of good ideas is that they lead to further insights and unexpected explanations for related (but not necessarily focal) phenomena, that is, they are not just testable, but are also generative [89] (Table 1, criterion 8). Having said this, nascent hypotheses may not yet have had sufficient time or attention to generate deeper and unexpected insights, so failure to do so is not grounds for rejection of a scenario, even if alternatives might generate more excitement.

## 7. Evaluation of Scenarios

**Figure 2:**
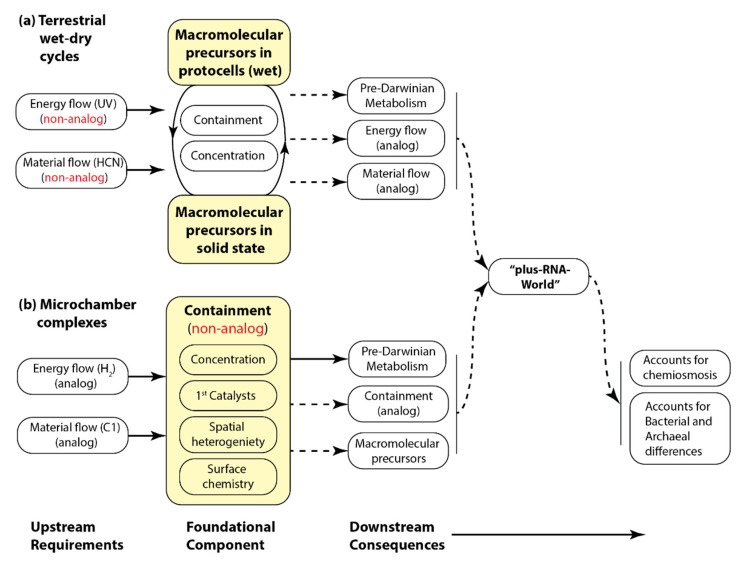
Structure of the (**a**) terrestrial wet–dry cycles [21] and (**b**) marine microchamber complex scenarios for the origin of life [12]. Terrestrial wet–dry cycles scenarios typically posit an energy source (UV light) and source materials (HCN) not used by life today, and thus are designated non-analog. Similarly, the microchamber complex scenario posits that initial containment was provided by the microchamber walls, not used by life today, and so is also designated as non-analog. The dashed lines indicate steps that are not (currently) well explained by the scenario.

Recognizing that we don’t yet understand how life originated, and that the complexity of the problem and richness of the relevant data and ideas make comparison among scenarios difficult, we use the criteria in Table 1 to evaluate the most comprehensive scenarios proposed to date. The scenarios we examine fall into two broad classes, the marine alkaline hydrothermal group, and the terrestrial wet–dry cycling group (see Section 1.2). Figure 2 diagrams their domains of explanatory power.

Given the highly structured, long-lived, and numerous microchambers at the alkaline hydrothermal vents we (currently) favor this venue for the origin of life, which makes sense of several stages in the history of life—it provides insights from the earliest stages of prebiotic synthesis, to the dawn of life, to the nature of LUCA, to the origin of the chemiosmotic production of ATP, and, finally, to the emergence of Bacteria and Archaea (see Section 8). This is not to say, however, that some other scenario(s), might not be able to make a more compelling case, but simply that we feel that none have yet done so across all these criteria (Table 1). 

Perhaps the weakest component of the alkaline hydrothermal vent microchamber complex scenario is its difficulty in accounting for the pre-Darwinian synthesis of polypeptides and, especially, polynucleotides (RNA and DNA), given that their polymerizations are dehydration reactions, which are hard to envision in the aqueous microchambers [90]. We return to this issue shortly. Others have criticized variants of the microchamber complex scenario that argue for the pH gradient, and thus chemiosmosis, as a driving force in the pre-Darwinian stages of life (for example, see [21,91,92,93], but see also [94]). However, with a steady source of H_2_ from the underlying serpentinization, it does not seem that the proton gradient is needed as an energy source for the scenario to work (although it has been argued to play a role in the reduction of CO_2_ [95]). Thus, in our reading of the scenario, the use of the pH gradient as a metabolic energy source was not relevant until after the “plus-RNA-World”, nor was it an absolute requirement.

We note that the alkaline hydrothermal vent microchamber complex scenario is closely similar to the alkaline hydrothermal vent green rust mound scenario [17], an offshoot scenario motivated by the identification of a microenvironment associated with alkaline hydrothermal vents where dehydration reactions might occur. Both scenarios are also very similar to the alkaline hydrothermally charged sediment scenario, where containment may have been achieved via mineral gels, which may offer a solution to performing dehydration reactions [18]. However, none of these latter scenarios makes as strong a contact with the top-down data criteria (Table 1, points 5–7), largely because they lack the long-lived stable cell-like structures provided by the microchamber complexes, although it is not impossible that the smaller spaces between sedimentary grains or green rust lamellae served the same purpose. 

The other major class of scenarios center on the wet–dry terrestrial geothermal venues [20,21,22], especially at their edges [21] where wet–dry prebiotic syntheses can occur [47]. One of the primary motivations for these scenarios is the observation that phosphorylation reactions are thermodynamically unfavorable in solution [22,90,96,97]. These scenarios do not make strong contact with the criteria from top-down data (Table 1, points 5–7), but are not fundamentally at odds with them either, although the scenarios do not lend themselves easily to an explanation of the differences in the cell walls, membranes, and independently derived mechanisms for motility of the Archaea and Bacteria. In terms of criterion 2 (the probability of life under the scenario), the biggest concern stems from the ephemerality of the setting (the edges of geothermal hot springs or small ponds) and the slowness of the fundamental dynamics of the system, where each round of innovation requires a wet–dry cycle, with the constant collapse and re-encapsulation of the protocells. Nonetheless, the proposed multiple rounds of innovation, selection, reconsolidation, followed by the re-aggregation of the contents of the protocells during the drying phase, has an appealing generative quality it to it (Table 1, criterion 8). For example, the constant re-building of the protocell and its contents is in principle consistent with the accumulation of integrated complexity (Table 1, criterion 3), and is also consistent with the bottom-up principle of steady regeneration, a point only rarely emphasized [21,90]. 

Finally, we note that terrestrial scenarios face the possibility that while there was continental crust by at least 4 billion years ago [98,99] that the origin of emergent continental crust, that is terrestrial habitat, may have been as late as 3.5 billion years ago [98,100], but see [101], or even from 2.5 to 3 billion years ago [102]. However, others argue for terrestrial habitat much earlier [103]; similar to the study of the origin of life, understanding the early history of the Earth is also very difficult due to a lack of data, and there are still many points of disagreement [99,104]. 

### 7.1. Primary Weakness of the Marine Microchamber Scenario—Dehydration Reactions in the Deep

The terrestrial scenarios draw strength from the high-yield experiments using UV-activated electrons and wet–dry cycling to generate amino acids, lipids, and nucleotides [47]. The fact that polymerizations that produce polypeptides and polynucleotides are dehydration reactions suggests that the making of these molecules is not well suited to a submarine environment [90]. Thus, in many ways the most important differences between the two scenarios boils down to water. 

### 7.2. Water

There are three points relevant to the issue of water and dehydration reactions we wish to make, and then a fourth in Section 7.3. First, as we all know, water is essential for life [49,105,106,107,108,109]. Thus, an open question for the terrestrial scenarios is how did the synthesis of biomolecules via wet–dry cycles become transferred to permanently water-based cells, that is, how was the transition made from non-analog energy and material flow to analog flows, given the level of complexity inferred to have developed at the protocell stage prior to the advent of ribozymes and enzymes (criterion 7). That is, it seems that the terrestrial scenarios also have to deal with the synthesis of polymers based on dehydration reactions in an aqueous environment prior to the evolution of ribozymes. 

Second, the behavior of water is complex in small volumes [106,110], especially ones that have high concentrations of other molecules [106,111], that is, it is still unclear what water’s properties really are, beyond the fact that its activity is significantly reduced in these settings [49,109]. Like the water in cells today, the water inside the vent microchambers is unlikely to have had the properties of dilute aqueous solutions typically used in in vitro experiments [111], where the concentrations of biomolecules are typically a 10th to 100th that observed in eukaryotic cells [112]. In many respects, the prebiotic aqueous environment inside the vent microchambers probably resembled the protoplasm of a modern cell. The rich pre-Darwinian milieu within the microchambers may well have meant that dehydration reactions occurred readily, permitting polymer formation, especially given the molecular complexity of pre-Darwinian life (see Section 4.5). In this context, we again note that dehydration reactions do occur today in abundance within the overall aqueous environment of the living cell, within relatively dry compartments, such as peptidyl transferase reactions within the ribosome. It seems premature to write the entire scenario off, given the complexity of the problem. 

Finally, the microchamber complexes have enormous surface areas, with extensive solid-water interfaces. Initial prebiotic synthetic reactions may well have occurred at these surfaces, for example, the initial reactions catalyzed by transition metal sulfides, where the surfaces may have facilitated the concentration and alignment of reactants [12,31,32,64]. With the advent of a progressively more complex pre-Darwinian biochemistry, for example, via the deposition of lipids and proteinoids on the surfaces, dehydration reactions may well have occurred on these microchamber surfaces. We note in this context that bio-molecular synthesis can occur at hydrophobic air–water interfaces [96], producing polypeptides [113] and promoting complex polynucleotide chemistry [114]—it is possible that the initial synthesis of biopolymers may have occurred at the surfaces of micro-bubbles within the microchamber complexes. But it is also possible that polynucleotide synthesis occurred late in the pre-Darwinian phase (see Section 5), rendering the need for their prebiotic synthesis moot.

### 7.3. A Hybrid Scenario?

Finally, we offer the possibility that the green rust variant of the alkaline hydrothermal vent scenario [17] might offer the solution to where the initial dehydration reactions occurred, as well as other prebiotic syntheses [115,116,117], within the nano-confinement between the iron (oxy)hydroxide layers, for example, if they were deposited in the sediments under the microchamber complexes, that is, upstream of the microchambers. Then those molecules would have been fed into the microchambers. We have advocated for the importance of spatial heterogeneity from the outset (Table 1, criterion 4); perhaps it began by including more than just the microchambers.

## 8. The Microchamber Complex Scenario

We now outline the microchamber complex scenario (Table 2), including its strengths in providing a venue with the physical and chemical environment needed for the development of life from its prebiotic stages to the emergence of free-living cells, including key steps required for the development of biochemical pathways, especially the need for spatial heterogeneity, easy access to prebiotic catalysts, and the early development of a rich and evolving community of diverse organic molecules. 

Finally, we posit that after the transition from ribozymes to enzymes that the evolution of ATP synthase was the accelerant that led to the fragmentation of the common biology inside the ancestral microchamber complex, thus establishing LUCA and the concomitant divergence of the Bacteria and Archaea. 

### 8.1. A Little History

The first deep sea hydrothermal vents of the ~700 now known [118], the ‘black smokers’, were discovered in 1977 on the mid-ocean ridge of the East Pacific Rise [119]. Lying far below the photic zone at a depth of 2500 m the research team was stunned to find a rich community of organisms. While there is a long history behind the idea of life originating at marine hydrothermal vents (see mini-review in [28]), once the energy source of the community was elucidated, chemosynthesis [120], it was suggested that ‘black smokers’ might have been the cradle of life [121]. However, the acidity and high temperatures, up to 300 °C, were not conducive to the synthesis of organic molecules [122,123] and enthusiasm for the idea faded. 

In 1983 fossil vent chimneys, approximately 360 million years old, were discovered in Ireland [124] with associated minerals indicative of cooler conditions. This led Russell et al. [13] to hypothesize that this type of hydrothermal vent would be an attractive venue for life’s origin, especially once he realized that the fossil chimneys consisted of a ‘honeycomb’ of microchambers [125]. 

In 2000, Kelly and colleagues [126] discovered close analogs of these chimneys at the ‘Lost City’ hydrothermal vent field with an alkaline vent flux of pH from 9 to 11 and moderate temperatures (40–75 °C) flowing slowly through the structures. The key difference between the black smokers and the alkaline chimneys is that the alkaline chimneys form some distance from the mid-ocean ridge by the process of serpentinization, the interaction of sea water via fissures in the seafloor and mantle rock in the underlying oceanic core complex [127,128,129]. The return of the warm mineral-charged water to the open ocean precipitates the formation of the honeycombed vent chimneys, the microchamber complexes [130,131].

### 8.2. The Alkaline Vent Microchamber Complexes—Megacities 

At ‘Lost City’ the venting process results in structures 60 m tall and 100 m across [126,132], riddled with sub-millimeter-sized microchambers, varying in volume from cubic microns to cubic millimeters. A few chambers have thick walls effectively isolating their interiors, but most are relatively porous. The total density of microchambers, if one assumes an average length of 500 μm and a diameter of 100 μm, is 10^11^ microchambers per cubic meter, with most microchambers readily communicating with the vent flux [133], but with varying degrees of isolation and connection to each other—each vent spire is effectively a megacity of tiny connected microchambers.

### 8.3. Suitable Boundary Conditions

From the outset, the microchamber walls would have provided an effective delineation of inside from outside (Table 2, property 1), with a permeability suitable for the steady energy and material flows required from life’s earliest pre-Darwinian stages to the emergence of free-living cells (Table 1, criterion 1). This scenario suggests the microchamber walls are the homologs of the prokaryotic cell walls and membranes. However, as noted above (Section 4.2.), while the microchamber walls served as structural supports under this scenario, they likely became quickly coated with organic molecules, thus beginning to function as membranes early in the process of life’s origin. The hydrothermal vent flux provides a steady flow of abundant chemical energy (H_2_) driven by serpentinization, and materials (single carbon molecules), which flow through the microchambers to finally exit into the open ocean, carrying away waste [14,15] (Table 2, properties 2 and 3). 

The vent flux, temperature, redox, and pH gradients associated with the microchamber complexes satisfy the steady state dynamics upon which all life depends, constituting one of the most attractive features of the microchamber scenario (see [134]). Moreover, the fact that the oldest carbon fixation pathway known among organisms today, the acetyl-CoA pathway [135,136], uses H_2_ as an electron donor and CO_2_ as an electron acceptor means [137] that one does not need to hypothesize a shift in the energy resources used in prebiotic synthesis as sophistication increased toward crown group life, unlike the terrestrial scenarios which require a weaning from the non-analog UV light and HCN as life developed.

The microchamber scenario also provides a simple mechanism for concentrating precursor molecules. The temperature gradient across the tiny microchambers would lead to the concentration of molecules via thermophoresis [138,139,140] (Table 2, property 4) (but see [141] for an alternative view), while the small volumes of the microchambers mean that high concentrations could be achieved even with small absolute amounts of precursor molecules (Table 2, property 5). 

### 8.4. The Origin of Life in the Microchamber Complexes as a Massively Parallel Process

One of the criteria for evaluating OoLoE scenarios is how likely life is under each of them (Table 1, criterion 2), with, all else being equal, geographically extensive and long-lived venues being favored over more ephemeral venues. 

In the case of the alkaline hydrothermal vents, the Lost City hydrothermal field is relatively long-lived, greater than 120,000 years [142]. This is on the order of 10^12^ s, a vast time compared to chemical reaction times which are on the order of milliseconds to seconds (or longer) (Table 2, property 6). 

A second consideration is the sheer number of microchambers in each hydrothermal field. With a density of ~10^11^ microchambers per cubic meter in a microchamber complex (Table 2, property 7) and with a volume of up to 10 m^3^, there can be in the order of 10^12^ microchambers per individual vent in each hydrothermal field. Further, each complex probably has many relatively independent ‘sectors’ of microchambers as the water flows radially from the core of the complex. Thus, each microchamber complex can be viewed as constituting a huge number of independent sets of experiments. Moreover, each hydrothermal field typically consists of multiple active vents. 

We can also ask how many microchamber complexes there were prior to life’s origin. Today the alkaline hydrothermal vents are found where mantle rock reaches sufficiently shallow depths for serpentinization to occur [143], including close to ultra-slow (<20 mm/year) to slow seafloor spreading ridges (20–50 mm/year), which constitute a significant proportion of the 60,000 km of mid-ocean ridges today [144]. In the Hadean it appears that plate tectonics as we know it may not have existed [98,104,145,146], and that there was a much greater exposure of mantle rock on the sea floor [147,148]. Today alkaline hydrothermal vents are one of the most geographically extensive of all proposed venues for the origin of life—in the Hadean they may have been vastly more numerous on a seafloor with its abundant Al-depleted komatiites [148].

The fact that even within one vent microchamber complex there is a vast number of microchambers means that there would have been an enormous range of subtly different physical and chemical environments—the full range of microchambers on the sea floor over the prebiotic history of the young Earth could thus be viewed as a massively parallel reactor system poised to generate life. 

#### Multiple Origins of Life

**Figure 3:**
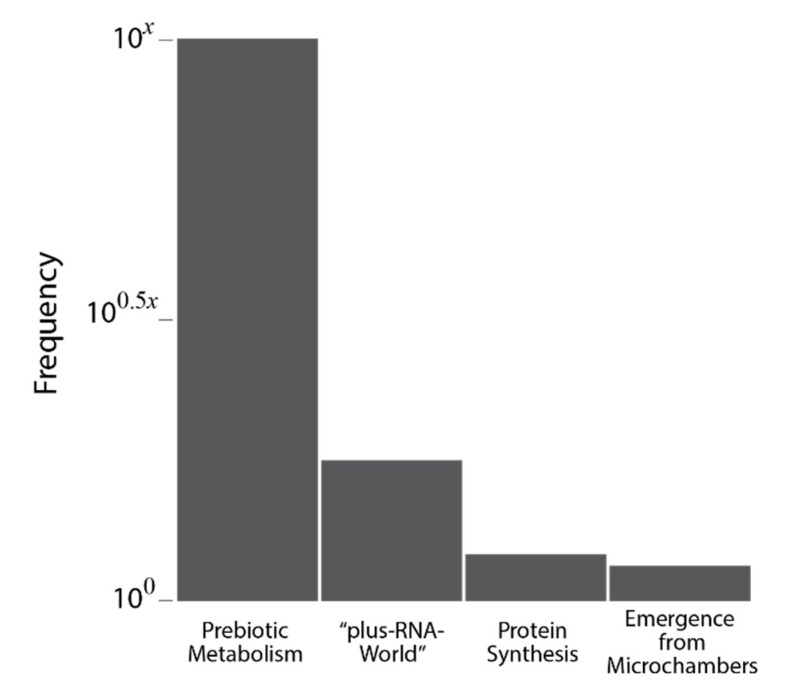
Conceptual histogram expressing our sense that the pre-Darwinian stage of life emerged a great many times, while relatively few made the difficult transition to the “plus-RNA-World”, with even less achieving protein synthesis, followed by perhaps an easier transition to emergence from the microchambers as free-living cells. Reflecting our ignorance, we have assigned frequencies in terms of 10*^x^*, where the value of *x* is unknown. If *x* = 12, then the scaling of the histogram indicates 1,000,000,000,000 origins of prebiotic metabolism, 1000 “plus-RNA-Worlds”, 10 origins of protein synthesis, and six emergences, which implies the extinction of four of them.

While we have no data bearing directly on the number of times life originated, we simply note that the magnitude of the numbers outlined above suggest that life may have originated a great number of times, with an unknown decrease in frequency at each of the major stages in development (Figure 3). In fact, given the pervasiveness of extinction on geologic timescales [149], it has been argued that it is likely that life must have originated multiple times to have had a reasonable chance of leaving descendants today [150].

**Figure 4:**
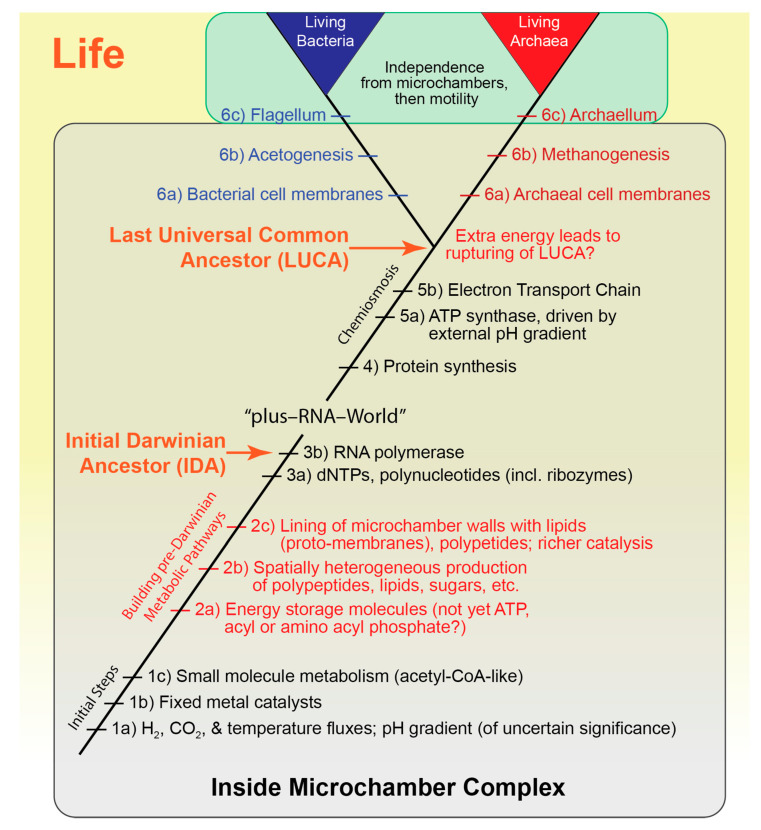
Hypothesized sequence of major events in the origin and early evolution of life within the ancestral microchamber complex, with a focus on the pre-Darwinian stages, and on the emergence of the Bacteria and Archaea. Innovations in red are emphasized in the text. Most of the events depicted occurred within the confines of the vent microchambers; only at the very top do Bacteria and Archaea emerge as free-living cells. The developments prior to IDA are relatively slow due to limited energy and nonspecific catalysts. Beginning with IDA, Darwinian evolution accelerated the rate of change, while between IDA and LUCA ribozymes were replaced by enzymes. Eventually energy from the natural proton gradient, harnessed by ATP synthase, provided an energy source in addition to the H_2_ from serpentinization. Following LUCA, the progenitors of Bacteria and Archaea evolved within the microchamber complex prior to their emergence as free-living cells. Life as defined here includes both stem and crown group life (see Figure 1). Note we have not depicted the likely extensive role of horizontal transfer in these early phases of life’s history (but see Figure 1).

We now outline the major events in the evolution of life under the microchamber complex scenario, starting with the earliest prebiotic stage and continuing to the emergence of the already well-differentiated stem groups of the Bacteria and Archaea (Figure 4).

### 8.5. The Microchamber Complex Scenario—Initial Steps

The earliest stages of prebiotic life under the microchamber complex scenario have been well described, including the central importance of the containment the microchambers would have provided, the abundance of H_2_, single carbon molecules (including CO_2_) and temperature and pH gradients, along with plausible models of how the first prebiotic metabolism might have evolved [12,14,64,135,137] (Figure 4, step 1a). The open/closed structure of the microchambers provides the isolation required for innovation coupled with connections enabling products and processes to be shared. Within in a single microchamber complex there may have been millions of relatively independent experiments (as would be the case for the hydrothermally-charged sediments scenarios [18,31], and the wet–dry cycles scenario [21]). We feel the rigidity of the vent microchamber complex structure was particularly important, providing a static constancy for the prebiotic chemistry until it was replaced by the dynamic stability of biomolecular systems. The role(s) of the pH gradient at these earliest stages remains an open question [91,94], and given that ATP synthase could not have evolved until after the origin of protein synthesis, it seems highly unlikely that chemiosmosis was critically important in the early phases of life, even if the pH gradient played a role in the assimilation of CO_2_ and other aspects of the prebiotic and early pre-Darwinian chemistry (e.g., see [32]).

In almost all scenarios for the origin of life, both terrestrial and marine, the initial catalysts are assumed to have involved transition metals particularly Fe and Ni with sulphur (Table 2, property 9) [16,51,135,137,151,152,153], although more recently a wider net has been cast [154,155]. Their antiquity is supported by the prevalence of ferredoxins, with their Fe(Ni)S centers, in metabolic pathways [16,51,135,152] (Figure 4, step 1b). The fact that the oldest carbon fixation pathway known among organisms today, the acetyl-CoA pathway, uses H_2_ as an electron donor and CO_2_ as an electron acceptor, suggests that an acetyl-CoA-like pathway was present from the earliest stages [135,136,137] (Figure 4, step 1c).

### 8.6. Building Biochemical Pathways

Organisms can be characterized by their network of biochemical pathways, including the relevant reactants and catalysts. With microchambers providing cell-like conditions (Table 2, properties 1–5; Figure 4, steps 1a–c), the next step in the emergence of life was establishing the biosynthetic pathways for more complex molecules, which typically require linking exergonic reactions with endergonic reactions, spatial heterogeneity, and mobile catalysts.

#### 8.6.1. Linking Exergonic and Endergonic Steps—Energy Storage Molecules

As is well understood, reactions will only occur spontaneously if exergonic, yet almost all biochemical synthesis pathways have endergonic steps, requiring the input of free energy. A key step during the pre-Darwinian era was the coupling of exergonic and endergonic reactions [156] via soluble energy storage molecules. The earliest phase of prebiotic synthesis in the microchambers likely occurred on the microchamber walls, catalyzed by embedded transition metal complexes, for example, Fe(Ni)S [137,152]. However, with fixed catalysts the coupling of an endergonic reaction at one site to an exergonic reaction at another site on the wall would be difficult [157], but the coupling of exergonic and endergonic reactions is much easier with energy intermediate molecules that can accept energy from an exergonic reaction and then transfer it later to another reaction elsewhere. In modern biology adenosine triphosphate (ATP) and its relatives serve as these energy intermediates [158]. However, in the prebiotic microchambers simpler molecules probably functioned as vehicles for intermediate energy storage, for example, thioesters [159], reduced ferredoxins [160,161], or perhaps acyl or amino acyl phosphate [135,162] (Figure 4, step 2a). The conclusion that ATP was not used is supported by the observation that removal of ATP from the food-set of organic co-factors has no impact on the inferred core metabolism of LUCA [74]. The rise to dominance of nucleotide triphosphates as energy intermediate molecules in modern biology was probably related to their role as substrates for nucleic acid synthesis.

#### 8.6.2. Spatial Heterogeneity

As noted in Section 3.2, spatial heterogeneity is crucial to cellular function in living organisms, and thus we posit that chemical inhomogeneity was necessary from the earliest phases of life’s development (Table 1, criterion 4). Spatial heterogeneity is inherent in the networks of microchambers of alkaline hydrothermal vents, and this may have provided the chemical and physical heterogeneity critical for life’s origin (Table 2, property 10; Figure 4, step 2b). Given the way they form, virtually all the microchambers in a complex have some degree of connection with adjacent microchambers, forming interconnected networks that share reactants and products as the vent flux flows through the complexes. The thermal, redox, and pH gradients between the hot vent flux and the frigid surrounding sea water varies among microchambers. Given that the vent flux flows radially outwards, traversing perhaps 10^3–4^ microchambers before reaching the edge of the complex and the open ocean, it seems likely that the chambers in the rind (the outer surface of the vent complex) would experience the steepest gradients where the vent flux meets the open ocean. The enormous number of microchambers constitutes a vast array of interacting environments for the initial lithochemical reactions, through to the transition to living systems, and on to free-living cells [15,163]. 

#### 8.6.3. Likely Complexity of the Prebiotic Molecular Community

The microchambers provide enclosed spaces where organic materials can accumulate, including complex macromolecules such as polypeptides, complex lipids, polysaccharides, etc. Many of these macromolecules have an affinity for surfaces and likely accumulated on the microchamber walls, both enhancing the catalytic activity of the embedded transition metals as well as modifying the permeability of these walls [12,31,32,64]. Macromolecules lining the walls would have moderated the movement of molecules into and out of the microchambers [64,164]), serving as proto-membranes (Figure 4, step 2c). The lined microchamber would have helped control the composition of the molecular milieu within the microchambers permitting molecules to accumulate to high concentrations, which would have fostered the production of sophisticated molecules. Note that under this hypothesis, the macromolecule lined microchambers are viewed as homologues of the cell (see also Section 8.6.4), rather than small coacervate protocells lying within the microchambers [105,134]. 

Long before the transition to Darwinian life, it seems likely that the vent microchambers were the sites of heterogeneous organic molecular syntheses where high concentrations of complex organic molecules, including polymers, could accumulate [107,165,166,167]. The prebiotic chemistry was complex, potentially with components without analogs in modern biology, but that nonetheless may have played an important role in the emergence of life. We note that, if this scenario is accurate, it may be challenging to develop realistic experimental analogs of the complex pre-Darwinian stage of life.

#### 8.6.4. Multiple Microchambers May Be the Homolog of the Cell

If indeed spatial heterogeneity was essential for life to emerge, then it is possible that the first entity to have a stable biochemistry, and thus the first entity that might be viewed as being alive, extended over several or even a great many microchambers, and thus that such a collection of microchambers is the homolog of the living cell. Thus, it is quite possible that the standard emphasis on cells as the fundamental unit of life should not be extended back to life’s origin, less it distract us from a broader view of life [69].

#### 8.6.5. Transition from Fixed Inorganic Catalysts to Mobile Organic Catalysts

As noted in Section 8.6.3 above, if catalysts are immobile, embedded in the microchamber walls, the products of one reaction cannot be used efficiently as substrates for following reactions as the reaction products will diffuse away before they can participate in the next reaction. This limitation effectively precludes the formation of biochemical pathways, and thus catalysts with fixed locations severely limited the potential development of prebiotic chemical systems [157]. In contrast mobile catalysts permit reactions to be linked into chains, forming biochemical pathways. Here, we simply note that while ribozymes, catalytic RNAs, are typically viewed as the first mobile catalysts, it seems likely that proteinoids, random polypeptide chains, complexed with transition metals may in fact have been the first mobile catalysts (Figure 4, step 2c) [166].

### 8.7. The Transition to Darwinian Life—From Nucleotides to Ribozymes to RNA Polymerase

While the prebiotic vent microchambers were likely havens for the synthesis of complex organic molecules, the resulting community of molecules did not reproduce and thus did not evolve in a Darwinian sense [39,40]. Thus, the advent of the first ribozyme RNA polymerases heralded the beginning of Darwinian life, initiating the “plus-RNA-World” (Figure 4, steps 3a,b) [168,169], a term we use given the growing consensus of the complexity of pre-Darwinian life (Section 4.6).

We note that one of the major challenges of experimental approaches to understanding the origin of life is the in vitro synthesis of ribozyme RNA polymerases, which has now been achieved [170], although still with a significant loss of catalytic activity after replication, thus still evoking the concerns of Eigen’s error threshold [166]. 

Ribozyme RNA polymerases capable of the production of accurate full-length products [170,171] would have initiated a community of evolving RNA molecules with a wide variety of functions. Despite the likely importance of pre-Darwinian small molecule and polypeptide catalysts, once evolving ribozymes appeared their capacity for replication meant that they would have come to dominate the molecules in the microchambers, while the operation of natural selection building on the already complexly functioning metabolism would most likely have led to a steady increase in the functionality of the ribozymes. Thus, first Darwinian life would have become dependent on RNAs as catalysts. Ribozymes are sufficiently versatile catalysts that they likely expanded the networks of reactions to the point that the “plus-RNA-World” may well have rivaled modern biochemical pathways in their complexity [168,169,172], given the top-down data that indicate that LUCA was very complex.

### 8.8. From Ribozymes to Enzymes

Once a propagating evolving community of RNA molecules was established, a ribozyme capable of catalyzing the formation of polypeptides, one with peptidyl transferase activity, would have been favored by selection, even though initially the primitive peptidyl transferase probably would have produced polypeptides with random sequences. The descendent of this early ribozyme is the large subunit ribosomal RNA (LSU rRNA) molecule which harbors the peptidyl transferase today [173], one of the few [174] catalytic remnants of the “plus-RNA-World” [168].

While still shrouded in mystery [175,176,177], the consequences of the development of protein synthesis were enormous, with proteins serving a wide range of functions, for example, structural support, transport, signaling, etc. Proteins as catalysts, enzymes, are such superior catalysts to RNAs that virtually all ribozyme catalysts in the biochemical pathways developed during the era of the “plus-RNA-World” we expect were rapidly replaced by enzymes (Figure 4, step 4). 

RNAs have two weaknesses as catalysts compared with polypeptides. First, catalysts need to have a stable conformation so that reactions can be consistently catalyzed [178], but RNAs tend to be floppy, in large part because all their component parts are hydrophilic. Second, RNAs only have four residues, and with the purines, A and G, being almost alike as are the pyrimidines, U and C, and given the similarity between purines and pyrimidines, they provide very little raw material for making sophisticated compartments for reactants—the fact that the structure of polynucleotides is ideal for duplex formation and thus replication means that their structure is not ideal for forming catalytic structures. Generating hydrophobic regions is difficult with the hydrophilic bases—thus forming even a modest catalytic conformation requires a very long RNA (and even in the case of the ribosome, the long catalytic LSU rRNA is stabilized by the ribosomal proteins).

Compared to RNAs, polypeptide chains readily form stable structures with hydrophobic interiors and many nooks and crannies [179], well suited to bringing reactants into close proximity with specificity and precision, orientations that promote specific reactions. Even generic proteinoids with no specific sequence, due to their wide variety of amino acid side chain residues, can form globular structures which provide hydrophobic regions that can facilitate numerous reactions that will not proceed in an aqueous environment [179,180]. 

#### 8.8.1. Protein Synthesis Greatly Accelerated the Rate of Evolution 

While the steps involved in the evolution of the complex process of translation are not well understood [175,176,177], but see [181], it is clear that the catalytic capacity afforded by protein synthesis dramatically increased rates of reaction. Thus, the rate at which energy could be extracted from the environment and the rate of evolution increased, probably by many orders of magnitude. 

#### 8.8.2. ATP Synthase 

Among the many novel protein complexes generated after the advent of translation was ATP synthase, a veritable molecular machine [182,183] by which most organisms today use to generate their ATP using energy from the flow of protons through the complex, that is, via chemiosmosis [184]. Explaining the development of chemiosmosis is a major challenge for OoLoE scenarios. However, the microchamber complex scenario provides a solution, viz: the nascent ATP synthase (however the complex evolved) used the naturally occurring proton gradient across the microchamber walls (Table 2, property 11; Figure 4, step 5a) [88,185], which greatly increased the energy available to the microchamber molecular communities. This explanation for why organisms use chemiosmosis is a most appealing feature of the microchamber complex scenario [186] (Table 1, criterion 8). 

One of the concerns of the hypothesis that the naturally occurring pH gradient provided the environmental opportunity for the evolution of ATP synthase is that the pH gradient across any one microchamber was probably quite small, and that ATP synthase works best with thin and impermeable (and thus well developed) cell membranes [91]. However, given that the emergence of ATP synthase requires the existence of protein synthesis, we surmise that ATP synthase emerged in an already highly sophisticated molecular community, in the microchambers with the steepest pH gradients, presumably those in the outer rind of the microchamber complexes (see Section 8.6.2), and quite possibly with already well developed proto-membranes. 

The evolution of ATP synthase was a key innovation, permitting the vent creature to tap the natural vent proton gradient, thus adding a new external energy source to the H_2_ derived from serpentinization. As we explain in Section 8.9 we posit that this additional energy harnessed by the ATP synthase increased the rate of evolution within the molecular community to the point that the community fragmented, which led to the development of the separate archaeal and bacterial cell lineages.

#### 8.8.3. The Electron Transport Chain

Independent of the development of ATP synthase was the evolution of the electron transport chain (ETC), which in today’s cells generates the proton gradient that runs ATP synthase. Energy generated by the passage of electrons via the chain of proteins, that constitute the ETC, pumps protons across a membrane to create the proton gradient. The advent of the ETC, which presumably evolved for some other purpose than driving ATP synthase (perhaps to help control the interior pH), was thus able to provide a proton gradient independent of the natural vent proton gradient (Figure 4, step 5b). The steps involved in the evolution of this process are not well understood, and likely involved the pumping of other ions, especially Na^+^, across the nascent cell membranes [16,32,59]. In this context, the discovery of a shallow water alkaline hydrothermal setting with both pH and Na^+^ gradients in Icelandic coastal waters [84] suggests the possibility that ATP synthase might have initially run on a sodium gradient, rather than a proton gradient (or both), had life originated in a similar environment. The microchamber complex scenario elegantly posits that ATP synthase evolved in response to a natural proton (or Na^+^) gradient and that the evolution of the ETC, whatever its initial function, meant that ATP synthase could then function in the absence of a natural gradient [16,32,59].

#### 8.8.4. LUCA

Analysis of the living biota indicates that the last universal common ancestor (LUCA) had all the attributes listed above—all extant organisms share the same machinery for protein synthesis [187], the same genetic code, tRNAs, ribosomal RNAs, and many proteins, as well as ATP synthase, and the electron transport chain. As whole genomes continue to pour in [188,189], and with steady bioinformatics innovation [189,190,191,192], we will continue to see refinements in the attributes of LUCA, a task we do not undertake here. 

### 8.9. The Increase in Available Energy Ruptured the Microchamber Molecular Community, Thereby Creating LUCA

Prior to LUCA the evolving molecular community inside the ancestral microchamber complex shared a common biology, which implies that the rate of dispersal of advantageous innovations throughout the microchamber complex, at least those that have survived to the present, was able to keep up with the rate of innovation. The evidence for this is simple, the complexity inferred to have been present in LUCA. But we also know that at some point the bacterial and archaeal lineages began to diverge (Section 4.2, Section 4.3 and Section 8.9.1, Figure 4). Under the microchamber complex scenario this divergence occurred within the ancestral microchamber complex [50,64]. So, what might have caused a once universal set of biochemistries inside the ancestral microchamber complex to then diverge? We posit that it was the exploitation of the natural vent proton gradient by ATP synthase that provided an additional energy resource sufficient to accelerate the rate of evolution to the point where the molecular community inside the ancestral microchamber complex ruptured, that is, with the increase in usable energy (in the form of ATP) innovations occurred faster than they could be dispersed throughout the complex, resulting in the separate histories we see in the Archaea and Bacteria. Whatever caused the split, it in effect created LUCA, marking the end of stem-group life.

#### 8.9.1. Independent Emergence of Bacteria and Archaea

The conclusion that the Bacteria and Archaea diverged within the ancestral micro-chamber complex is based on the evidence that they independently acquired necessities for existence as free-living cells (see Section 4.2). Additional differences between the two lineages include their metabolisms—stem group Archaea were methanogens while stem group Bacteria were generally acetogens (Figure 4, steps 6b) [12,16,59]. Moreover, there appear to be no ion-pumping proteins that are universal across the tree of life, suggesting that the co-option of the ETC system to replace the external pH gradient occurred independently in each lineage [163]. 

Thus, the microchamber complex scenario accounts for both the similarities and differences between the two prokaryotic lineages alive today, the Bacteria and Archaea (Table 2, point 12), an unexpected outcome of a hypothesis that began by considering the earliest steps in the origin of life as we know it [13] (Table 1, criterion 8).

#### 8.9.2. A Note on the Role of DNA

The centrality of DNA as the information storage molecule for life today is common knowledge, but that role emerged well after the IDA. Here, we simply point out that while RNA has a special role in the origin of Darwinian life, several authors have proposed that RNA and DNA may well have emerged simultaneously, perhaps forming chimeric RNA/DNA structures (see review in [68]), with a range of nucleotides having prebiotic non-coding roles [193]. We note that while catalytic DNAs have been synthesized [194], there are no DNA catalysts in living cells. Only later did DNA’s stability lend selective advantage to its use as the primary information storage molecule [195]. The late role of DNA as the primary information storage molecule finds support in the observation that Bacteria and Archaea use different processes and enzymes to initiate the replication of their DNA [58], suggesting that the primary role of DNA in information storage may have arisen twice [50], well after the origin of protein synthesis.

## 9. Quo Vadis?

For the reasons outlined above we currently favor a late-ATP synthase version of the alkaline hydrothermal vent microchamber complex scenario for the origin of life, with the possibility that green rust processes played a role. The microchambers would appear to be an ideal venue for the transition for prebiotic to living systems, and from there to the launching of free-living cells, the Bacteria and Archaea, that were to thrive in ways that would have seemed impossible at the time of their emergence. 

One might ask, should we abandon other scenarios, for example, the terrestrial wet–dry cycling scenarios? The answer is an unequivocal “No”! We have so much to learn, and we do not know from where key breakthroughs or unexpected perspectives will come. Only the future will determine how our collective understanding will advance. Science can be viewed as making maps into the unknown, while objectivity can be viewed as the effectiveness of those maps as a means of conveying understanding to others [196]. We feel the microchamber hypothesis represents the best map currently available, but it is still crude and may have major flaws—the true map may surprise us all.

## Figures and Tables

**Table 1:** Criteria for evaluating origin of life on Earth (OoLoE) scenarios, beyond satisfying the laws of chemistry and physics.

Criterion
*Bottom-up criteria—initial conditions*
1. OoLoE initiated with permeable containment in an environment with pervasive and steady energy and material flows.
2. Probability of life—cradle of life was long-lived and/or geographically extensive.
3. Integration of developing metabolic pathways began from the outset.
4. Spatial heterogeneity was present from the outset.
*Accommodation of top-down data*
5. Full cellular autonomy and motility occurred post-LUCA.
6. Minimization of the number of inferred non-analog to analog transitions (see text).
7. Origin of complex integrated metabolism before ribozymes and enzymes, made possible by mineral and metal ion catalysts and energy storage molecules.
*Additional Criterion*
8. Scenario offers unexpected insights and generates a wide range of predictions.

**Table 2:** Properties of the alkaline hydrothermal vent microchamber complexes conducive to the building of a plausible scenario for the origin of life, from the stage of prebiotic chemistry to the origin of crown group Archaea and Bacteria.

Property	Attractiveness of the Microchamber Complexes
*Cell-Like Boundary Conditions*	
1. Delineation of inside and outside	Microchamber walls provided protection from the external environment, while being permeable to energy and material flows.
2. Energy flow	The chemical energy flowing through the microchambers, H_2_, is used by the most primitive carbon fixation pathway, the acetyl-CoA pathway.
3. Material flow	Abundant single-carbon molecules, including CO_2_ which is used in the acetyl-CoA pathway, are available.
4. Ease of concentration	The tiny chambers in conjunction with the thermal gradient concentrates molecules (thermophoresis).
5. Little precursor material needed	The tiny spaces mean that relatively little prebiotic material would be needed to produce high concentrations of prebiotic molecules.
*Probability of Life Originating*	
6. Temporally long-lived	Hydrothermal fields are estimated to have a lifetime of ~10^5^ years (10^12^ s). On the Hadean seafloor they may have been even longer lived.
7. Numerous microchambers in a complex	The sub-millimeter size of the chambers translates to some 10^11^ interconnected microchambers/m^3^ per vent.
8. Many microchamber complexes	Hadean serpentinization may have occurred over much of the seafloor, thus over the (perhaps) 10s to 100s of millions of years between the formation of the sea floor and the origin of life, the number of potential cradles of life may have been enormous.
*Establishing Biochemical Pathways*	
9. Ample prebiotic catalysts	Abundant Fe(Ni)S minerals present, some of which would have served as excellent prebiotic catalysts.
10. Varying degrees of separation/connectedness/spatial heterogeneity	The 10^11^ variably interconnected microchambers/m^3^ translates into a vast range of subtly different geochemical conditions and spatial heterogeneity, facilitating complex prebiotic and early biotic synthesis.
*Accounts for Top-Down Observations*	
11. Accompanying pH gradient offers explanation for the origin of chemiosmosis	Chemiosmotic generation of ATP is central to most organisms’ energy production but is hard to explain. The presence of an extremal pH (and possibly Na^+^) gradient across the microchamber complex means that the flow of protons was integral to the proposed cradle of life and means that ATP synthase could have functioned without the electron transport chain.
12. Explains Bacterial and Archaeal differences	Includes the evidence that the two lineages became fully cellularized and mobile indpendently (see Section 8.9).

## Data Availability

Not applicable.

## References

[1] Preiner M., Asche S., Becker S., Betts H.C., Boniface A., Camprubi E., Chandru K., Erastova V., Garg S.G., Khawaja N. (2020). The future of origin of life research: Bridging decades-old divisions. Life.

[2] Russell M.J. (2021). The “Water Problem”(sic), the Illusory Pond and Life’s Submarine Emergence—A Review. Life.

[3] Miller S.L. (1953). A production of amino acids under possible primitive earth conditions. Science.

[4] Miller S.L., Urey H.C. (1959). Organic compound synthesis on the primitive Earth. Science.

[5] Zaug A.J., Cech T.R. (1980). In vitro splicing of the ribosomal RNA precursor in nuclei of *Tetrahymena*. Cell.

[6] Guerrier-Takada C., Gardiner K., Marsh T., Pace N., Altman S. (1983). The RNA moiety of ribonuclease P is the catalytic subunit of the enzyme. Cell.

[7] Kitadai N., Maruyama S. (2018). Origins of building blocks of life: A review. Geosci. Front..

[8] Becker S., Schneider C., Crisp A., Carell T. (2018). Non-canonical nucleosides and chemistry of the emergence of life. Nat. Commun..

[9] Chandru K., Jia T.Z., Mamajanov I., Bapat N., Cleaves H.J. (2020). Prebiotic oligomerization and self-assembly of structurally diverse xenobiological monomers. Sci. Rep..

[10] Benner S.A., Bell E.A., Biondi E., Brasser R., Carell T., Kim H.-J., Mojzsis S.J., Omran A., Pasek M.A., Trail D. (2020). When Did Life Likely Emerge on Earth in an RNA-First Process?. ChemSystemsChem.

[11] Martin W., Baross J., Kelley D., Russell M.J. (2008). Hydrothermal vents and the origin of life. Nat. Rev. Microbiol..

[12] Martin W., Russell M.J. (2007). On the origin of biochemistry at an alkaline hydrothermal vent. Philos. Trans. R. Soc. B Biol. Sci..

[13] Russell M.J., Hall A.J., Cairns-Smith A.G., Braterman P.S. (1988). Submarine hot springs and the origin of life. Nature.

[14] Russell M.J., Hall A.J., Martin W. (2010). Serpentinization as a source of energy at the origin of life. Geobiology.

[15] Russell M.J., Barge L.M., Bhartia R., Bocanegra D., Bracher P.J., Branscomb E., Kidd R., McGlynn S., Meier D.H., Nitschke W. (2014). The drive to life on wet and Icy Worlds. Astrobiology.

[16] Sousa F.L., Thiergart T., Landan G., Nelson-Sathi S., Pereira I.A.C., Allen J.F., Lane N., Martin W.F. (2013). Early bioenergetic evolution. Philos. Trans. R. Soc. B Biol. Sci..

[17] Russell M.J. (2018). Green rust: The simple organizing ‘seed’ of all life?. Life.

[18] Westall F., Hickman-Lewis K., Hinman N., Gautret P., Campbell K.A., Bréhéret J.G., Foucher F., Hubert A., Sorieul S., Dass A.V. (2018). A Hydrothermal-Sedimentary Context for the Origin of Life. Astrobiology.

[19] Wächtershäuser G. (2007). On the chemistry and evolution of the pioneer organism. Chem. Biodivers..

[20] Mulkidjanian A.Y., Bychkov A.Y., Dibrova D.V., Galperin M.Y., Koonin E.V. (2012). Origin of first cells at terrestrial, anoxic geothermal fields. Proc. Natl. Acad. Sci. USA.

[21] Damer B., Deamer D. (2020). The hot spring hypothesis for an origin of life. Astrobiology.

[22] Sasselov D.D., Grotzinger J.P., Sutherland J.D. (2020). The origin of life as a planetary phenomenon. Sci. Adv..

[23] Brasier M.D., Matthewman R., McMahon S., Wacey D. (2011). Pumice as a remarkable substrate for the origin of life. Astrobiology.

[24] Brasier M.D., Matthewman R., McMahon S., Kilburn M.R., Wacey D. (2013). Pumice from the ∼3460Ma Apex Basalt, Western Australia: A natural laboratory for the early biosphere. Precambrian Res..

[25] Hansma H.G. (2017). Better than membranes at the origin of life?. Life.

[26] Bernhardt H.S. (2019). Making molecules with clay: Layered double hydroxides, pentopyranose nucleic acids and the origin of life. Life.

[27] Rasmussen B., Muhling J.R., Fischer W.W. (2021). Greenalite Nanoparticles in Alkaline Vent Plumes as Templates for the Origin of Life. Astrobiology.

[28] Camprubí E., de Leeuw J.W., House C.H., Raulin F., Russell M.J., Spang A., Tirumalai M.R., Westall F. (2019). The Emergence of Life. Space Sci. Rev..

[29] Darwin Correspondence Project, “Letter no. 7471”. https://www.darwinproject.ac.uk/letter/DCP-LETT-7471.xml.

[30] Peretó J., Bada J.L., Lazcano A. (2009). Charles Darwin and the origin of life. Orig. Life Evol. Biosph..

[31] Wächtershäuser G. (2010). Chemoautotrophic origin of life: The iron-sulfur world hypothesis. Geomicrobiology: Molecular and Environmental Perspective.

[32] Sojo V., Herschy B., Whicher A., Camprubí E., Lane N. (2016). The Origin of Life in Alkaline Hydrothermal Vents. Astrobiology.

[33] Blackmond D.G. (2020). Autocatalytic Models for the Origin of Biological Homochirality. Chem. Rev..

[34] Gould S.B. (2018). Membranes and evolution. Curr. Biol..

[35] Marshall C.R., Valentine J.W. (2010). The importance of preadapted genomes in the origin of the animal bodyplans and the Cambrian explosion. Evolution.

[36] Donoghue P.C.J. (2005). Saving the stem group—a contradiction in terms?. Paleobiology.

[37] Yarus M. (2011). Getting past the RNA world: The initial Darwinian ancestor. Cold Spring Harb. Perspect. Biol..

[38] Morowitz H.J. (1968). Energy Flow in Biology.

[39] Darwin C. (1859). On the Origin of Species.

[40] Mayr E. (1991). One Long Argument: Charles Darwin and the Genesis of Modern Evolutionary Thought.

[41] Trifonov E.N. (2011). Vocabulary of definitions of life suggests a definition. J. Biomol. Struct. Dyn..

[42] Bartlett S., Wong M.L. (2020). Defining lyfe in the universe: From three privileged functions to four pillars. Life.

[43] Astrobiology at NASA—About Life Detection. https://astrobiology.nasa.gov/research/life-detection/about/.

[44] Thornhill R.H., Ussery D.W. (2000). A classification of possible routes of Darwinian evolution. J. Theor. Biol..

[45] Lyon A.S., Peeples W.B., Rosen M.K. (2021). A framework for understanding the functions of biomolecular condensates across scales. Nat. Rev. Mol. Cell Biol..

[46] Cohan M.C., Pappu R.V. (2020). Making the Case for Disordered Proteins and Biomolecular Condensates in Bacteria. Trends Biochem. Sci..

[47] Patel B.H., Percivalle C., Ritson D.J., Duffy C.D., Sutherland J.D. (2015). Common origins of RNA, protein and lipid precursors in a cyanosulfidic protometabolism. Nat. Chem..

[48] Clark B.C., Kolb V.M. (2020). Macrobiont: Cradle for the origin of life and creation of a biosphere. Life.

[49] do Vieira A.N., Kleinermanns K., Martin W.F., Preiner M. (2020). The ambivalent role of water at the origins of life. FEBS Lett..

[50] Koonin E.V., Martin W. (2005). On the origin of genomes and cells within inorganic compartments. Trends Genet..

[51] Weiss M.C., Sousa F.L., Mrnjavac N., Neukirchen S., Roettger M., Nelson-Sathi S., Martin W.F. (2016). The physiology and habitat of the last universal common ancestor. Nat. Microbiol..

[52] Weiss M.C., Preiner M., Xavier J.C., Zimorski V., Martin W.F. (2018). The last universal common ancestor between ancient Earth chemistry and the onset of genetics. PLoS Genet..

[53] Gogarten J.P., Deamer D. (2016). Is LUCA a thermophilic progenote?. Nat. Microbiol..

[54] Berkemer S.J., McGlynn S.E., Berkemer S.J., McGlynn S.E. (2021). A new analysis of archaea-bacteria domain separation: Variable phylogenetic distance and the tempo of early evolution. Mol. Biol. Evol..

[55] Orgel L.E. (1968). Evolution of the genetic apparatus. J. Mol. Biol..

[56] Morowitz H.J. (1993). Beginnings of Cellular Life: Metabolism Recapitulates Biogenesis.

[57] Leduc S. (1911). The Mechanism of Life.

[58] Bleichert F., Botchan M.R., Berger J.M. (2017). Mechanisms for initiating cellular DNA replication. Science.

[59] Sojo V., Pomiankowski A., Lane N. (2014). A Bioenergetic Basis for Membrane Divergence in Archaea and Bacteria. PLoS Biol..

[60] Koga Y., Kyuragi T., Nishihara M., Sone N. (1998). Did archaeal and bacterial cells arise independently from noncellular precursors? A hypothesis stating that the advent of membrane phospholipid with enantiomeric glycerophosphate backbones caused the separation of the two lines of descent. J. Mol. Evol..

[61] Lombard J., López-García P., Moreira D. (2012). The early evolution of lipid membranes and the three domains of life. Nat. Rev. Microbiol..

[62] Vollmer W., Bertsche U. (2008). Murein (peptidoglycan) structure, architecture and biosynthesis in Escherichia coli. Biochim. Biophys. Acta Biomembr..

[63] Albers S.V., Meyer B.H. (2011). The archaeal cell envelope. Nat. Rev. Microbiol..

[64] Martin W., Russell M.J. (2003). On the origins of cells: A hypothesis for the evolutionary transitions from abiotic geochemistry to chemoautotrophic prokaryotes, and from prokaryotes to nucleated cells. Philos. Trans. R. Soc. B Biol. Sci..

[65] Chen I.A., Walde P. (2010). From self-assembled vesicles to protocells. Cold Spring Harb. Perspect. Biol..

[66] Beeby M., Ferreira J.L., Tripp P., Albers S.V., Mitchell D.R. (2020). Propulsive nanomachines: The convergent evolution of archaella, flagella and cilia. FEMS Microbiol. Rev..

[67] Harrison S.A., Lane N. (2018). Life as a guide to prebiotic nucleotide synthesis. Nat. Commun..

[68] Yadav M., Kumar R., Krishnamurthy R. (2020). Chemistry of Abiotic Nucleotide Synthesis. Chem. Rev..

[69] Muchowska K.B., Varma S.J., Moran J. (2020). Nonenzymatic Metabolic Reactions and Life’s Origins. Chem. Rev..

[70] Hordijk W., Steel M. (2004). Detecting autocatalytic, self-sustaining sets in chemical reaction systems. J. Theor. Biol..

[71] Steel M., Hordijk W., Xavier J.C. (2019). Autocatalytic networks in biology: Structural theory and algorithms. J. R. Soc. Interface.

[72] Hordijk W., Kauffman S.A., Steel M. (2011). Required levels of catalysis for emergence of autocatalytic sets in models of chemical reaction systems. Int. J. Mol. Sci..

[73] Kauffman S.A. (1986). Autocatalytic sets of proteins. J. Theor. Biol..

[74] Xavier J.C., Hordijk W., Kauffman S., Steel M., Martin W.F. (2020). Autocatalytic chemical networks at the origin of metabolism. Proc. R. Soc. B Biol. Sci..

[75] Bowman J.C., Hud N.V., Williams L.D. (2015). The Ribosome Challenge to the RNA World. J. Mol. Evol..

[76] Frenkel-Pinter M., Haynes J.W., Mohyeldin A.M., Martin C., Sargon A.B., Petrov A.S., Krishnamurthy R., Hud N.V., Williams L.D., Leman L.J. (2020). Mutually stabilizing interactions between proto-peptides and RNA. Nat. Commun..

[77] Wheat C.G., McManus J., Mottl M.J., Giambalvo E. (2003). Oceanic phosphorus imbalance: Magnitude of the mid-ocean ridge flank hydrothermal sink. Geophys. Res. Lett..

[78] Albarède F., Blichert-Toft J. (2009). The terrestrial cradle of life. Orig. Life Self-Organ. Biol. Evol..

[79] Goldford J.E., Hartman H., Smith T.F., Segrè D. (2017). Remnants of an Ancient Metabolism without Phosphate. Cell.

[80] Rasmussen B., Muhling J.R., Suvorova A., Fischer W.W. (2021). Apatite nanoparticles in 3.46–2.46 Ga iron formations: Evidence for phosphorus-rich hydrothermal plumes on early Earth. Geology.

[81] Deamer D. (2017). The role of lipid membranes in life’s origin. Life.

[82] Szostak J.W., Bartel D.P., Luisi P.L. (2001). Synthesizing life. Nature.

[83] Jordan S.F., Rammu H., Zheludev I.N., Hartley A.M., Maréchal A., Lane N. (2019). Promotion of protocell self-assembly from mixed amphiphiles at the origin of life. Nat. Ecol. Evol..

[84] Price R., Boyd E.S., Hoehler T.M., Wehrmann L.M., Bogason E., Valtýsson H.P., Örlygsson J., Gautason B., Amend J.P. (2017). Alkaline vents and steep Na+ gradients from ridge-flank basalts-Implications for the origin and evolution of life. Geology.

[85] Dibrova D.V., Galperin M.Y., Koonin E.V., Mulkidjanian A.Y. (2015). Ancient systems of sodium/potassium homeostasis as predecessors of membrane bioenergetics. Biochemistry.

[86] Mulkidjanian A.Y., Galperin M.Y., Koonin E.V. (2009). Co-evolution of primordial membranes and membrane proteins. Trends Biochem. Sci..

[87] Mulkidjanian A.Y., Galperin M.Y., Makarova K.S., Wolf Y.I., Koonin E.V. (2008). Evolutionary primacy of sodium bioenergetics. Biol. Direct.

[88] Lane N., Allen J.F., Martin W. (2010). How did LUCA make a living? Chemiosmosis in the origin of life. BioEssays.

[89] Lakatos I., Lakatos I., Musgrave A. (1970). Falsification and the Methodology of Scientific Research Programmes. Criticism and the Growth of Knowledge.

[90] Sutherland J.D. (2017). Opinion: Studies on the origin of life-The end of the beginning. Nat. Rev. Chem..

[91] Jackson J.B. (2016). Natural pH Gradients in Hydrothermal Alkali Vents Were Unlikely to Have Played a Role in the Origin of Life. J. Mol. Evol..

[92] Jackson J.B. (2017). Ancient living organisms escaping from, or imprisoned in, the vents?. Life.

[93] Ross D.S. (2018). It is Neither Frankenstein Nor a Submarine Alkaline Vent, It is Just the Second Law. BioEssays.

[94] Lane N. (2017). Proton gradients at the origin of life. BioEssays.

[95] Hudson R., de Graaf R., Rodin M.S., Ohno A., Lane N., McGlynn S.E., Yamada Y.M.A., Nakamura R., Barge L.M., Braun D. (2020). CO2 reduction driven by a pH gradient. Proc. Natl. Acad. Sci. USA.

[96] Nam I., Lee J.K., Nam H.G., Zare R.N. (2017). Abiotic production of sugar phosphates and uridine ribonucleoside in aqueous microdroplets. Proc. Natl. Acad. Sci. USA.

[97] Sutherland J.D. (2016). The Origin of Life—Out of the Blue. Angew. Chem. Int. Ed..

[98] Hawkesworth C.J., Cawood P.A., Dhuime B. (2020). The Evolution of the Continental Crust and the Onset of Plate Tectonics. Front. Earth Sci..

[99] Harrison T.M. (2020). Hadean Earth.

[100] Flament N., Coltice N., Rey P.F. (2013). The evolution of the 87sr/86sr of marine carbonates does not constrain continental growth. Precambrian Res..

[101] Keller C.B., Harrison T.M. (2020). Constraining crustal silica on ancient Earth. Proc. Natl. Acad. Sci. USA.

[102] Johnson B.W., Wing B.A. (2020). Limited Archaean continental emergence reflected in an early Archaean ^18^O-enriched ocean. Nat. Geosci..

[103] Harrison T.M. (2009). The hadean crust: Evidence from >4 Ga zircons. Annu. Rev. Earth Planet. Sci..

[104] Arndt N.T., Nisbet E.G. (2012). Processes on the young earth and the habitats of early life. Annu. Rev. Earth Planet. Sci..

[105] Westall F., Brack A. (2018). The Importance of Water for Life. Space Sci. Rev..

[106] Ball P. (2008). Water as an active constituent in cell biology. Chem. Rev..

[107] Frenkel-Pinter M., Rajaei V., Glass J.B., Hud N.V., Williams L.D. (2021). Water and Life: The Medium is the Message. J. Mol. Evol..

[108] De Duve C. (1995). Vital Dust: Life as a Cosmic Imperative.

[109] Branscomb E., Russell M.J. (2019). On the beneficent thickness of water. Interface Focus.

[110] Ball P. (2008). Water: Water—An enduring mystery. Nature.

[111] Saha R., Pohorille A., Chen I.A. (2014). Molecular Crowding and Early Evolution. Orig. Life Evol. Biosph..

[112] Ellis R.J. (2001). Macromolecular crowding: An important but neglected aspect of the intracellular environment. Curr. Opin. Struct. Biol..

[113] Griffith E.C., Vaida V. (2012). In situ observation of peptide bond formation at the water-air interface. Proc. Natl. Acad. Sci. USA.

[114] Morasch M., Liu J., Dirscherl C.F., Ianeselli A., Kühnlein A., Le Vay K., Schwintek P., Islam S., Corpinot M.K., Scheu B. (2019). Heated gas bubbles enrich, crystallize, dry, phosphorylate and encapsulate prebiotic molecules. Nat. Chem..

[115] White L.M., Shibuya T., Vance S.D., Christensen L.E., Bhartia R., Kidd R., Hoffmann A., Stucky G.D., Kanik I., Russell M.J. (2020). Simulating serpentinization as it could apply to the emergence of life using the JPL hydrothermal reactor. Astrobiology.

[116] Barge L.M., Flores E., VanderVelde D.G., Weber J.M., Baum M.M., Castonguay A. (2020). Effects of Geochemical and Environmental Parameters on Abiotic Organic Chemistry Driven by Iron Hydroxide Minerals. J. Geophys. Res. Planets.

[117] Flores E., Martinez E., Rodriguez L.E., Weber J.M., Khodayari A., Vandervelde D.G., Barge L.M. (2021). Effects of Amino Acids on Phosphate Adsorption onto Iron (Oxy)hydroxide Minerals under Early Earth Conditions. ACS Earth Space Chem..

[118] Lu G.S., LaRowe D.E., Fike D.A., Druschel G.K., Gilhooly W.P., Price R.E., Amend J.P. (2020). Bioenergetic characterization of a shallow-sea hydrothermal vent system: Milos Island, Greece. PLoS ONE.

[119] Edmond J.M., Von Damm K.L., McDuff R.E., Measures C.I. (1982). Chemistry of hot springs on the East Pacific Rise and their effluent dispersal. Nature.

[120] Jannasch H.W. (1985). Review Lecture-The chemosynthetic support of life and the microbial diversity at deep-sea hydrothermal vents. Proc. R. Soc. London Ser. B. Biol. Sci..

[121] Baross J.A., Hoffman S. (1985). Submarine hydrothermal vents and associated gradient environments as sites for the origin and evolution of life. Orig. Life.

[122] Miller S.L., Bada J.L. (1988). Submarine hot springs and the origin of life. Nature.

[123] Bada J.L., Lazcano A. (2002). Some like it hot, but not the first biomolecules. Science.

[124] Boyce A.J., Coleman M.L., Russell M.J. (1983). Formation of fossil hydrothermal chimneys and mounds from Silvermines, Ireland. Nature.

[125] Russell M. (2006). Fist Life. Am. Sci..

[126] Kelley D.S., Karson J.A., Blackman D.K., Früh-Green G.L., Butterfield D.A., Lilley M.D., Olson E.J., Schrenk M.O., Roe K.K., Lebon G.T. (2001). An off-axis hydrothermal vent field near the mid-Atlantic ridge at 30° n. Nature.

[127] Whitney D.L., Teyssier C., Rey P., Roger Buck W. (2013). Continental and oceanic core complexes. Bull. Geol. Soc. Am..

[128] Ildefonse B., Blackman D.K., John B.E., Ohara Y., Miller D.J., MacLeod C.J., Abe N., Abratis M., Andal E.S., Andréani M. (2007). Oceanic core complexes and crustal accretion at slow-spreading ridges. Geology.

[129] Blackmann D.K., Karson J.A., Kelley D.S., Cann J.R., Früh-Green G.L., Gee J.S., Hurst S.D., John B.E., Morgan J., Nooner S.L. (2002). Geology of the Atlantis Massif (Mid-Atlantic Ridge, 30°N): Implications for the evolution of an ultramafic oceanic core complex. Mar. Geophys. Res..

[130] Cardoso S.S.S., Cartwright J.H.E. (2017). On the differing growth mechanisms of black-smoker and Lost City-type hydrothermal vents. Proc. R. Soc. A Math. Phys. Eng. Sci..

[131] Ludwig K.A., Kelley D.S., Butterfield D.A., Nelson B.K., Früh-Green G. (2006). Formation and evolution of carbonate chimneys at the Lost City Hydrothermal Field. Geochim. Cosmochim. Acta.

[132] Denny A.R., Kelley D.S., Früh-Green G.L. (2015). Geologic evolution of the Lost City Hydrothermal Field. Geochem. Geophys. Geosyst..

[133] Kelley D.S., Karson J.A., Früh-Green G.L., Yoerger D.R., Shank T.M., Butterfield D.A., Hayes J.M., Schrenk M.O., Olson E.J., Proskurowski G. (2005). A serpentinite-hosted ecosystem: The Lost City hydrothermal field. Science.

[134] Budin I., Szostak J.W. (2010). Expanding roles for diverse physical phenomena during the origin of life. Annu. Rev. Biophys..

[135] Martin W.F. (2020). Older Than Genes: The Acetyl CoA Pathway and Origins. Front. Microbiol..

[136] Varma S.J., Muchowska K.B., Chatelain P., Moran J. (2018). Native iron reduces CO_2_ to intermediates and end-products of the acetyl-CoA pathway. Nat. Ecol. Evol..

[137] Preiner M., Igarashi K., Muchowska K.B., Yu M., Varma S.J., Kleinermanns K., Nobu M.K., Kamagata Y., Tüysüz H., Moran J. (2020). A hydrogen-dependent geochemical analogue of primordial carbon and energy metabolism. Nat. Ecol. Evol..

[138] Baaske P., Weinert F.M., Duhr S., Lemke K.H., Russell M.J., Braun D. (2007). Extreme accumulation of nucleotides in simulated hydrothermal pore systems. Proc. Natl. Acad. Sci. USA.

[139] Salditt A., Keil L.M.R., Horning D.P., Mast C.B., Joyce G.F., Braun D. (2020). Thermal Habitat for RNA Amplification and Accumulation. Phys. Rev. Lett..

[140] Duhr S., Braun D. (2006). Thermophoretic depletion follows Boltzmann distribution. Phys. Rev. Lett..

[141] Joyce G.F., Szostak J.W. (2018). Protocells and RNA self-replication. Cold Spring Harb. Perspect. Biol..

[142] Ludwig K.A., Shen C.C., Kelley D.S., Cheng H., Edwards R.L. (2011). U-Th systematics and 230Th ages of carbonate chimneys at the Lost City Hydrothermal Field. Geochim. Cosmochim. Acta.

[143] Schrenk M.O., Brazelton W.J., Lang S.Q. (2013). Serpentinization, carbon, and deep life. Rev. Mineral. Geochem..

[144] Mullineaux L.S., Metaxas A., Beaulieu S.E., Bright M., Gollner S., Grupe B.M., Herrera S., Kellner J.B., Levin L.A., Mitarai S. (2018). Exploring the ecology of deep-sea hydrothermal vents in a metacommunity framework. Front. Mar. Sci..

[145] Sleep N.H. (2007). Plate Tectonics through Time. Treatise Geophys..

[146] Sleep N.H. (2010). The Hadean-Archaean Environment. Cold Spring Harb. Perspect. Biol..

[147] Davies G.F. (2007). Dynamics of the Hadean and Archaean Mantle. Dev. Precambrian Geol..

[148] Shibuya T., Yoshizaki M., Sato M., Shimizu K., Nakamura K., Omori S., Suzuki K., Takai K., Tsunakawa H., Maruyama S. (2015). Hydrogen-rich hydrothermal environments in the Hadean ocean inferred from serpentinization of komatiites at 300 °C and 500 bar. Prog. Earth Planet. Sci..

[149] Marshall C.R. (2017). Five palaeobiological laws needed to understand the evolution of the living biota. Nat. Ecol. Evol..

[150] Raup D.M., Valentine J.W. (1983). Multiple origins of life. Proc. Natl. Acad. Sci. USA.

[151] Wachtershauser G. (1990). Evolution of the first metabolic cycles. Proc. Natl. Acad. Sci. USA.

[152] Camprubi E., Jordan S.F., Vasiliadou R., Lane N. (2017). Iron catalysis at the origin of life. IUBMB Life.

[153] Bonfio C., Valer L., Scintilla S., Shah S., Evans D.J., Jin L., Szostak J.W., Sasselov D.D., Sutherland J.D., Mansy S.S. (2017). UV-light-driven prebiotic synthesis of iron-sulfur clusters. Nat. Chem..

[154] Zhao D., Bartlett S., Yung Y.L. (2020). Quantifying mineral-ligand structural similarities: Bridging the geological world of minerals with the biological world of enzymes. Life.

[155] Li Y., Kitadai N., Nakamura R. (2018). Chemical diversity of metal sulfide minerals and its implications for the origin of life. Life.

[156] Schäfer G., Engelhard M., Müller V. (1999). Bioenergetics of the Archaea. Microbiol. Mol. Biol. Rev..

[157] Wächtershäuser G. (1988). Before enzymes and templates: Theory of surface metabolism. Microbiol. Rev..

[158] Knowles J.R. (1980). Enzyme-catalyzed phosphoryl transfer reactions. Annu. Rev. Biochem..

[159] Semenov S.N., Kraft L.J., Ainla A., Zhao M., Baghbanzadeh M., Campbell V.E., Kang K., Fox J.M., Whitesides G.M. (2016). Autocatalytic, bistable, oscillatory networks of biologically relevant organic reactions. Nature.

[160] Müller V., Chowdhury N.P., Basen M. (2018). Electron bifurcation: A long-hidden energy-coupling mechanism. Annu. Rev. Microbiol..

[161] Herrmann G., Jayamani E., Mai G., Buckel W. (2008). Energy conservation via electron-transferring flavoprotein in anaerobic bacteria. J. Bacteriol..

[162] Whicher A., Camprubi E., Pinna S., Herschy B., Lane N. (2018). Acetyl Phosphate as a Primordial Energy Currency at the Origin of Life. Orig. Life Evol. Biosph..

[163] Lane N., Martin W.F. (2012). The origin of membrane bioenergetics. Cell.

[164] Segré D., Ben-Eli D., Deamer D.W., Lancet D. (2001). The Lipid World. Orig. Life Evol. Biosph..

[165] Rajamani S., Vlassov A., Benner S., Coombs A., Olasagasti F., Deamer D. (2008). Lipid-assisted synthesis of RNA-like polymers from mononucleotides. Orig. Life Evol. Biosph..

[166] Frenkel-Pinter M., Samanta M., Ashkenasy G., Leman L.J., Örlygsson J. (2020). Prebiotic Peptides: Molecular Hubs in the Origin of Life. Chem. Rev..

[167] Tran Q.P., Adam Z.R., Fahrenbach A.C. (2020). Prebiotic reaction networks in water. Life.

[168] Gilbert W. (1986). The RNA world. Nature.

[169] Pressman A., Blanco C., Chen I.A. (2015). The RNA world as a model system to study the origin of life. Curr. Biol..

[170] Tjhung K.F., Shokhirev M.N., Horning D.P., Joyce G.F. (2020). An RNA polymerase ribozyme that synthesizes its own ancestor. Proc. Natl. Acad. Sci. USA.

[171] Eigen M. (1971). Selforganization of matter and the evolution of biological macromolecules. Naturwissenschaften.

[172] Robertson M.P., Joyce G.F. (2012). The origins of the RNA world. Cold Spring Harb. Perspect. Biol..

[173] Ban N., Nissen P., Hansen J., Moore P.B., Steitz T.A. (2000). The complete atomic structure of the large ribosomal subunit at 2.4 Åresolution. Science.

[174] Fedor M.J., Williamson J.R. (2005). The catalytic diversity of RNAs. Nat. Rev. Mol. Cell Biol..

[175] Koonin E.V., Novozhilov A.S. (2017). Origin and Evolution of the Universal Genetic Code. Annu. Rev. Genet..

[176] Koonin E.V. (2017). Frozen accident pushing 50: Stereochemistry, expansion, and chance in the evolution of the genetic code. Life.

[177] Wolf Y.I., Koonin E.V. (2007). On the origin of the translation system and the genetic code in the RNA world by means of natural selection, exaptation, and subfunctionalization. Biol. Direct.

[178] Anfinsen C.B. (1973). Principles that govern protein folding. Science.

[179] Perutz M.F. (1992). Introductory lecture: What are enzyme structures telling us?. Faraday Discuss..

[180] Perutz M.F. (1993). Co-chairman’s remarks: Before the double helix. Gene.

[181] Petrov A.S., Gulen B., Norris A.M., Kovacs N.A., Bernier C.R., Lanier K.A., Fox G.E., Harvey S.C., Wartell R.M., Hud N.V. (2015). History of the ribosome and the origin of translation. Proc. Natl. Acad. Sci. USA.

[182] Boyer P.D. (2002). A research journey with ATP synthase. J. Biol. Chem..

[183] Grüber G., Manimekalai M.S.S., Mayer F., Müller V. (2014). ATP synthases from archaea: The beauty of a molecular motor. Biochim. Biophys. Acta Bioenerg..

[184] Mitchell P. (1961). Coupling of phosphorylation to electron and hydrogen transfer by a chemi-osmotic type of mechanism. Nature.

[185] Krissansen-Totton J., Arney G.N., Catling D.C. (2018). Constraining the climate and ocean pH of the early Earth with a geological carbon cycle model. Proc. Natl. Acad. Sci. USA.

[186] Lane N. (2010). Why are cells powered by proton gradients?. Nat. Educ..

[187] Bowman J.C., Petrov A.S., Frenkel-Pinter M., Penev P.I., Williams L.D. (2020). Root of the Tree: The Significance, Evolution, and Origins of the Ribosome. Chem. Rev..

[188] Zhang Z., Wang J., Wang J., Wang J., Li Y. (2020). Estimate of the sequenced proportion of the global prokaryotic genome. Microbiome.

[189] Koonin E.V., Makarova K.S., Wolf Y.I. (2021). Evolution of Microbial Genomics: Conceptual Shifts over a Quarter Century. Trends Microbiol..

[190] Mende D.R., Letunic I., Maistrenko O.M., Schmidt T.S.B., Milanese A., Paoli L., Hernández-Plaza A., Orakov A.N., Forslund S.K., Sunagawa S. (2020). ProGenomes2: An improved database for accurate and consistent habitat, taxonomic and functional annotations of prokaryotic genomes. Nucleic Acids Res..

[191] Tonkin-Hill G., MacAlasdair N., Ruis C., Weimann A., Horesh G., Lees J.A., Gladstone R.A., Lo S., Beaudoin C., Floto R.A. (2020). Producing polished prokaryotic pangenomes with the Panaroo pipeline. Genome Biol..

[192] Howe K.L., Contreras-Moreira B., De Silva N., Maslen G., Akanni W., Allen J., Alvarez-Jarreta J., Barba M., Bolser D.M., Cambell L. (2020). Ensembl Genomes 2020-enabling non-vertebrate genomic research. Nucleic Acids Res..

[193] Fialho D.M., Roche T.P., Hud N.V. (2020). Prebiotic Syntheses of Noncanonical Nucleosides and Nucleotides. Chem. Rev..

[194] Ponce-Salvatierra A., Wawrzyniak-Turek K., Steuerwald U., Höbartner C., Pena V. (2016). Crystal structure of a DNA catalyst. Nature.

[195] Leu K., Obermayer B., Rajamani S., Gerland U., Chen I.A. (2011). The prebiotic evolutionary advantage of transferring genetic information from RNA to DNA. Nucleic Acids Res..

[196] Polanyi M. (1958). Personal Knowledge: Towards a Post-Critical Philosophy.

